# Influência da Atorvastatina na Hiperplasia Intimal em Modelo Experimental

**DOI:** 10.36660/abc.20200518

**Published:** 2020-10-13

**Affiliations:** 1 Universidade Estadual Paulista Faculdade de Medicina de Botucatu Departamento de Clínica Médica BotucatuSP Brasil Departamento de Clínica Médica, Faculdade de Medicina de Botucatu, Universidade Estadual Paulista (Unesp), Botucatu, SP – Brasil

**Keywords:** Atorvastatina/prevenção e controle, Hiperplasia, Ratos, Doença Arterial Coronariana/fisiopatologia, Enxerto Vascular, Músculo Liso Vascular, Modelos Animais

Apesar do significativo avanço da biomedicina cardiovascular nos últimos anos, que proporcionou melhor entendimento da fisiopatologia da doença arterial coronariana (DAC) bem como sua prevenção e tratamento, a DAC ainda é responsável por considerável parcela de óbitos.^1^

A DAC é o resultado do acúmulo patológico de placas ateroscleróticas nas artérias coronárias que pode levar a oclusão e isquemia do tecido cardíaco. Entre os tratamentos utilizados para a DAC, podemos destacar o enxerto de veia (EV), tipo de intervenção cirúrgica para a revascularização do miocárdio. Entretanto, a longo prazo, ocorre alta taxa de obstrução dos enxertos venosos, com remodelação expansiva e aumento da deposição de lipoproteína de baixa densidade (LDL), que pode ocasionar hiperplasia intimal (HI), aterosclerose e trombose.^2^^,^^3^ A HI está intimamente relacionada à reestenose do EV e se inicia em resposta a determinado estresse, o qual desencadeia processo inflamatório e consequente disfunção endotelial com proliferação e migração de células do músculo liso vascular (CMLV).^4^^,^^5^

As estatinas são inibidores da enzima HMG-CoA (3-hydroxyl-3-methylglutaryl coenzyme A) responsável pela síntese de colesterol.^6^ Dentro desta classe medicamentosa, a atorvastatina é comumente utilizada na terapêutica de pacientes com hipercolesterolemia e aterosclerose e possui capacidade de diminuir níveis de lipídios, plaquetas e processo inflamatório, atenuando dessa forma a ocorrência de eventos cardiovasculares.^7^ Já foi demonstrado, por estudo experimental com modelo de lesão carotídea, que a atorvastatina é capaz de suprimir a HI, por diminuir os níveis de lipídios sanguíneos e o acúmulo intimal de CMLV.^8^ Outro estudo com modelo semelhante mostrou que a redução da hiperplasia neointimal foi devido ao aumento da apoptose das CMLV.^9^

O estudo publicado nos *Arquivos Brasileiros de Cardiologia* desta edição teve como objetivo avaliar se a atorvastatina inibe a HI de EV em ratos,^10^ já que são escassos os estudos que verificaram os efeitos das estatinas na reestenose do enxerto após revascularização do miocárdio. Esses pesquisadores observaram que o tratamento com atorvastatina por quatro semanas após o enxerto foi eficaz em reduzir a espessura intimal, demonstrada pela diminuição de CMLV utilizando o PCNA (antígeno nuclear de proliferação celular) e a α-SMA (actina-alfa de músculo liso) como indicadores de proliferação destas células.^10^ A curiosidade dos autores em avaliar o efeito da atorvastatina na hiperplasia celular é pertinente porque demonstraram o efeito inibidor dessa medicação na proliferação de CMLV pela primeira vez em modelo de EV experimental. Além disso, os autores constataram que o tratamento com atorvastatina diminuiu a fosforilação da p38MAPK no enxerto venoso.^10^ Alguns estudos já haviam mostrado que as estatinas são capazes de suprimir a fosforilação da via p38MAPK induzida pela angiotensina II em cultura de CMLV^11^ e que a inibição dessa via por infusão da angiotensina-(1-7) em enxerto experimental de veia jugular atenuou o remodelamento vascular.^12^ No entanto o mecanismo de ação da p38MAPK na HI ainda não tinha sido avaliado no modelo de EV.^10^ O envolvimento da p38MAPK na HI foi recentemente verificado por estudo em que os autores mostraram que em lesões carotídeas experimentais há diminuição da expressão de miR-451. Quando esse microRNA está altamente expresso em CMLV ocorre bloqueio da sinalização da p38MAPK e diminuição da migração dessas células para o local da injúria.^13^

A pesquisa em que se baseia esse minieditorial demonstrou que a atorvastatina diminuiu os níveis de fosforilação de p38MAPK, e os autores associam esse achado com a redução da proliferação das CMLV no enxerto venoso, fatores que provavelmente foram envolvidos com a atenuação da HI. Desta forma, os achados do presente estudo indicam a importância da utilização das estatinas na prevenção da reestenose em enxertos venosos, fornecendo base para estudos clínicos. Além disso, o grupo poderá elucidar futuramente os possíveis mecanismos moleculares envolvidos com os benefícios deste medicamento nesse modelo experimental.
