# Supplementary material for: Influência da Atorvastatina na Hiperplasia Intimal em Modelo Experimental
Source: Arq Bras Cardiol. 2020 Oct 13;115(4):637–8. [Article in Portuguese] doi: 10.36660/abc.20200518 (PMC8386986; doi:10.36660/abc.20200518)
Supplement: Translation

# Translation: Influence of Atorvastatin on Intimal Hyperplasia in the Experimental Model

**Affiliations:** 1 BotucatuSP Brazil Departamento de Clínica Médica, Faculdade de Medicina de Botucatu, Universidade Estadual Paulista (Unesp), Botucatu, SP - Brazil

**Keywords:** Atorvastatin/prevention and control, Hyperplasia, Rats, Coronary Artery Dsease/physiopathology, Muscle, Smooth, Vascular, Models, Animal

Despite the significant advance in cardiovascular biomedicine in recent years, which provided a better understanding of the pathophysiology of coronary artery disease (CAD) as well as its prevention and treatment, this disease is still responsible for considerable mortality.^1^

The CAD results from the pathological accumulation of atherosclerotic plaques in the coronary arteries that can lead to their occlusion and ischemia of the cardiac tissue. Among the treatments used for CAD, stands out the venous graft (VG), a type of surgical intervention for coronary artery bypass grafting. However, in the long term, there is a high rate of obstruction of VG, with expansive remodeling and increased deposition of low-density lipoprotein (LDL), which can cause intimal hyperplasia (IH), atherosclerosis and thrombosis.^2^^,^^3^ The IH is closely related to restenosis of the VG and begins in response to certain stress, which triggers inflammatory process and consequent endothelial dysfunction with proliferation and migration of vascular smooth muscle cells (VSMC).^4^^,^^5^

Statins are inhibitors of the enzyme HMG-CoA (3-hydroxyl-3-methylglutaryl coenzyme A), which is responsible for the synthesis of cholesterol.^6^ Within this drug class, atorvastatin is commonly used in the treatment of patients with hypercholesterolemia and atherosclerosis and can decrease levels of lipids, platelets, and inflammatory processes, thus attenuating the occurrence of cardiovascular events.^7^ It has been demonstrated by an experimental study using a carotid lesion model that atorvastatin is capable of suppressing IH by decreasing blood lipid levels and VSMC accumulation in the intima.^8^ Another study with a similar model showed that the reduction in neointimal hyperplasia was due to increased VSMC apoptosis.^9^

The study published in the *Arquivos Brasileiros de Cardiologia* of this edition aimed to assess whether atorvastatin inhibits IH in rat vein graft^10^ since few studies verified the effects of the statins on VG restenosis after coronary artery bypass grafting. These researchers observed that treatment with atorvastatin for four weeks after the VG was effective in reducing the intimal thickness, demonstrated by the decrease in VSMC using PCNA (nuclear cell proliferation antigen) and α-SMA (α-smooth muscle actin) as proliferation indicators of these cells.^10^ The authors’ curiosity in evaluating the effect of atorvastatin on cell hyperplasia is relevant because they demonstrated the inhibitory effect of this medication on the VSMC proliferation for the first time in an experimental VG model. Besides, the authors found that treatment with atorvastatin decreased p38MAPK phosphorylation in the VG tissue.^10^ Some studies have been shown that statins are capable of suppressing p38MAPK pathway phosphorylation induced by angiotensin II in VSMC cultures^11^ and that inhibition of this pathway by angiotensin-(1-7) infusion in an experimental jugular vein graft attenuated vascular remodeling.^12^ However, the mechanism of action of p38MAPK in IH in the VG model had not yet been evaluated.^10^ The p38MAPK involvement in IH has recently been verified by a study in which the authors showed that in experimental carotid lesions, there is decreased expression of miR-451. When this microRNA is highly expressed in VSMC, it occurs blockage of p38MAPK signaling and decreased migration of these cells to the injury site.^13^

The research on which this short editorial is based demonstrated that atorvastatin decreased the p38MAPK phosphorylation, and the authors associate this finding with reduced proliferation of VSMC in the VG, factors that were probably involved with the attenuation of IH. Thus, the findings of the present study indicate the importance of using statins to prevent restenosis in VGs, providing a basis for clinical studies. Also, the group will be able to elucidate in the future the possible molecular mechanisms involved with the benefits of this drug in this experimental model.
